# German value sets for the EORTC QLU-C10D, a cancer-specific utility instrument based on the EORTC QLQ-C30

**DOI:** 10.1007/s11136-019-02283-w

**Published:** 2019-09-04

**Authors:** Georg Kemmler, Eva Gamper, Virginie Nerich, Richard Norman, Rosalie Viney, Bernhard Holzner, Madeleine King, the European Organisation for Research and Treatment of Cancer (EORTC) Quality of Life Group

**Affiliations:** 1 Division of Psychiatry I, Department of Psychiatry, Psychotherapy and Psychosomatics, Medical University of Innsbruck, Innsbruck, Austria; 2 Division of Psychiatry II, Department of Psychiatry, Psychotherapy and Psychosomatics, Medical University of Innsbruck, Innsbruck, Austria; 3 Department of Pharmacy, University Hospital of Besançon, Besançon, France; 4 University Bourgogne Franche-Comté, INSERM, EFS BFC, UMR1098, Interactions Hôte-Greffon-Tumeur/Ingénierie Cellulaire et Génique, Besançon, France; 5 School of Public Health, Curtin University, Perth, Australia; 6 Centre for Health Economics Research and Evaluation (CHERE), UTS Business School, University of Technology Sydney (UTS), Sydney, NSW Australia; 7 School of Psychology, Faculty of Science, University of Sydney, Sydney, NSW 2006 Australia; 8 Sydney Medical School, Faculty of Medicine, University of Sydney, Sydney, NSW Australia

**Keywords:** Utility weights, Discrete choice experiment, EORTC, QLQ-C30, QLU-C10D, Germany

## Abstract

**Purpose:**

The EORTC QLU-C10D is a new multi-attribute utility instrument derived from the EORTC QLQ-C30, a widely used cancer-specific quality of life questionnaire. It covers ten dimensions: physical, role, social, emotional functioning, pain, fatigue, sleep, appetite, nausea, and bowel problems. To allow national health attitudes to be reflected, country-specific valuations are being performed by collaboration of the Multi-Attribute Utility Cancer (MAUCa) Consortium and the EORTC. The purpose of this paper is to provide German value sets (utility weights) for the QLU-C10D.

**Methods:**

Valuations were run in a web-based setting in two general population samples of approximately 2000 adults in total. As the German version of the QLQ-C30 is presently undergoing a revision of the wording of one response category, valuations for both the current and the new version were performed (Germany 1 and 2). Utilities were elicited using a discrete choice experiment (DCE). Data were analyzed by conditional logistic regression and mixed logits.

**Results:**

Completion rates were 88.3% (1002/1135) and 90.4% (1016/1124) for Germany 1 and Germany 2 valuations, respectively. Dimensions with the largest impact on utility weights were, in this order: physical functioning, pain, role functioning, social functioning and nausea (same ordering for both German versions). Several violations of the logical ordering of levels were observed for Germany 1; this was largely improved for Germany 2.

**Conclusion:**

This study established German utility weights for the cancer-specific utility instrument QLU-C10D.

## Introduction

To inform rational decision making in health care, the results of economic evaluations have become increasingly important in the past decades. Cost utility analyses (CUA) have been recommended as the method of choice by various health authorities, such as NICE in the UK [1]. The primary outcome in a CUA is incremental cost per quality-adjusted life year (QALY). To estimate QALYs, life years are weighted by the utility of the health state experienced, where a value of 1 characterizes full health and 0 indicates a health state as poor as being dead.

There are various methods to obtain the required utilities for CUAs. One approach that has increasingly gained popularity is the use of multi-attribute utility instruments (MAUI) [2]. Widely used MAUIs include the EQ-5D [3] and the SF-6D [4]. The main advantage of MAUIs is that once utility weights have been determined for the set of health states covered, these weights can be applied in future CUAs without any further valuations.

MAUIs also provide a link between health economics and health-related quality of life (HRQOL) research, as they are frequently applied for the assessment of HRQOL. However, the most widely used MAUIs are generic utility instruments and thus somewhat limited in their coverage of HRQOL domains that may be relevant for decision making in specific patient populations.

Recently, *a cancer*-*specific* MAUI, the EORTC QLU-C10D, was developed as a collaboration of the Multi-Attribute Utility in Cancer (MAUCa) Consortium and the European Organisation for Research and Treatment of Cancer (EORTC) Quality of Life Group [5]. As a first step, the EORTC QLU-C10D, health state classification system was set up based on ten key dimensions of the EORTC QLQ-C30 [6], one of the most widely used cancer-specific quality of life (QOL) questionnaires. In a second step, country-specific utility weights (or value sets) for the QLU-C10D are being determined via valuation studies in general population samples in various countries. As health care systems and community attitudes towards health and illness vary across countries, the availability of country-specific value sets is essential.

Regarding the appropriate target group for the elicitation of health utilities, it should be noted that there is often a discrepancy between general population and patient preferences [7] and that ideally both perspectives should be taken into account [8]. In the present paper, we adopt the usual approach taken in health economics that CUAs are performed to guide societal decisions and hence should be primarily based on general population valuations [9].

In the first country-specific value set for the QLU-C10D, for Australia [10], discrete choice experiments (DCE) were used to elicit utilities, as this approach is considered advantageous in terms of comprehensibility and ease of application compared to classical approaches, such as the time trade-off method or the standard gamble [11, 12]. Subsequent QLU-C10D valuation studies for other countries are using the same methodology as the Australian study to facilitate comparability across countries.

The present paper deals with the determination of utility weights for Germany, as the first country within a series of QLU-C10D valuations in Europe. Valuations for several other European countries are underway. It should be noted that the German health authority, the Institute for Quality and Efficiency in Health Care (IQWiG), takes a critical attitude towards CUAs and recommends alternative strategies for economic evaluation of new treatments [13]. This controversy has been discussed in the literature [14, 15]. CUAs have nonetheless been performed in Germany despite the skeptical view of the IQWiG [16].

The German version of the QLQ-C30 is presently undergoing a revision of the wording of one of its response categories (level 3, “quite a bit”) as findings of a cross-national investigation demonstrated that the original German wording of this category was associated with a considerably lower severity level compared to the English label [17]. As QLU-C10D utilities are available not only for future CUAs but also for post hoc analyses of formerly conducted studies that used the QLQ-C30, determination of utility weights for both versions of the QLQ-C30 is necessary.

Thus, the principal aims of this paper are as follows:

1. Determination of general population utility weights for the German version of the QLU-C10D by means of the established DCE method approach, both for the old and the new wording of the response categories.
2. Comparison of the utility weights of the two German versions (old and new response wording) of the QLU-C10D. We hypothesized that utility weights for the response level “quite a bit” would differ significantly between the two versions.

## Methods

### EORTC QLQ-C30 and QLU-C10D

The EORTC QLQ-C30 [6] is a widely used cancer-specific QOL instrument. It comprises five functioning scales, nine symptom scales or items, and a global QOL scale. All functioning items and symptoms are rated on a 4-point Likert scale ranging from “not at all” to “very much.” The original German wording for the response category “quite a bit” (“mäßig”) was criticized for addressing a lower severity level than the corresponding English expression [17]. A new German version with revised wording for this response level (“ziemlich”) has almost completed the testing phase [18] and appears to be as a closer approximation of the severity level expressed by “quite a bit.” Hereafter, the two German versions will be abbreviated as QLQ-C30 German 1 (“mäßig”) and QLQ-C30 German 2 (“ziemlich”).

The QLU-C10D, the newly developed MAUI based on the QLQ-C30, consists of a health state classification system and an algorithm for the calculation of utilities for the health states defined by the system [5, 10]. The health states cover 10 key dimensions of the QLQ-C30: physical functioning, role functioning, social functioning, emotional functioning, pain, fatigue, sleep disturbances, appetite loss, nausea, and bowel problems. The severity of impairment in each dimension is expressed by the four categories used in the QLQ-C30: not at all, a little, quite a bit and very much. For use in the DCE, the survival time is also taken into account which can attain four distinct values: 1, 2, 5, and 10 years.

### DCE valuation task

Valuation of the German QLU-C10D was performed using the same methodology as employed in the Australian valuations. In particular, the same DCE design was used for elicitation of utilities. This was based on a total of 960 choice sets which were determined by methods of optimal design theory to maximize efficiency in estimating model parameters [19]. Each respondent had to complete 16 choice sets which were randomly selected from the 960. In each choice set, the respondent had to select one of two scenarios, A or B, each consisting of a health state defined by the ten attributes of the QLU-C10D and a survival time (Table 1). To keep the burden for the respondent at a manageable level, only five attributes in a choice set differed between scenarios (highlighted in yellow), whereas the severity level of the remaining attributes was kept equal. The order of the ten attributes was randomized for each respondent (and kept constant for each respondent across the 16 choice sets). The survival time was always presented last. For details regarding the DCE refer to the original paper [10]. An example of a choice set is shown in the Appendix (Fig. 5).

**Table 1:** Health state classification system of the QLU-C10D

Dimension	Level	Stem	Descriptor	QLQ-C30 item scores
Physical functioning^a,b^	1	You have…	No trouble taking a long walk outside of the house	Item 2 (long walk) = 1
	2		No trouble taking a short walk outside of the house, but at least a little trouble taking a long walk	Item 3 (short walk) = 1 AND Item 2 ≥ 2
	3		At least a little trouble taking a short walk outside of the house, and at least a little trouble taking a long walk	Item 3 = 2 AND Item 2 ≥ 2
	4		Quite a bit or very much trouble taking a short walk outside the house	Item 3 ≥ 3 AND Item 2 ≥ 2
Role functioning	1	You are limited in pursuing your work or other daily activities…	Not at all	Item 6 = 1
	2		A little	Item 6 = 2
	3		Quite a bit	Item 6 = 3
	4		Very much	Item 6 = 4
Social functioning^a^	1	Your physical condition or medical treatment interferes with your social or family life…	Not at all	Items 26 AND 27 = 1
	2		A little	Items 26 OR 27 = 2
	3		Quite a bit	Items 26 OR 27 = 3
	4		Very much	Items 26 OR 27 = 4
Emotional functioning	1	You feel depressed…	Not at all	Item 24 = 1
	2		A little	Item 24 = 2
	3		Quite a bit	Item 24 = 3
	4		Very much	Item 24 = 4
Pain	1	You have pain…	Not at all	Item 9 = 1
	2		A little	Item 9 = 2
	3		Quite a bit	Item 9 = 3
	4		Very much	Item 9 = 4
Fatigue	1	You feel tired…	Not at all	Item 18 = 1
	2		A little	Item 18 = 2
	3		Quite a bit	Item 18 = 3
	4		Very much	Item 18 = 4
Sleep	1	You have trouble sleeping…	Not at all	Item 11 = 1
	2		A little	Item 11 = 2
	3		Quite a bit	Item 11 = 3
	4		Very much	Item 11 = 4
Appetite	1	You lack appetite…	Not at all	Item 13 = 1
	2		A little	Item 13 = 2
	3		Quite a bit	Item 13 = 3
	4		Very much	Item 13 = 4
Nausea	1	You feel nauseated…	Not at all	Item 14 = 1
	2		A little	Item 14 = 2
	3		Quite a bit	Item 14 = 3
	4		Very much	Item 14 = 4
Bowel problems^a^	1	You…	do not have constipation or diarrhoea at all	Items 16 AND 17 = 1
	2		have a little constipation or diarrhoea	Items 16 OR 17 = 2
	3		have constipation or diarrhoea quite a bit	Items 16 OR 17 = 3
	4		have constipation or diarrhoea very much	Items 16 OR 17 = 4
Duration	1	You will live in this health state for…	1 year, and then die	Not applicable
	2		2 years, and then die	Not applicable
	3		5 years, and then die	Not applicable
	4		10 years, and then die	Not applicable

### Valuation survey

Separate valuation surveys for the two German versions of the QLU-C10D were run (denoted as Germany 1, Germany 2). Both surveys were administered online and consisted of the following parts: introduction, informed consent, information on age and sex for quota sampling (for Germany 2), the EORTC QLQ-C30, the DCE valuation task, four feedback questions on the DCE, further socio-demographic and basic clinical questions, EQ-5D-5L [20], and the Kessler K-10 mental health questionnaire [21]. Recruitment and data assessment were contracted to a company specialized in the conduct of DCEs which was successfully engaged for the Australian valuations.

The translation of the attributes for the DCE was taken from the validated German version of the QLQ-C30. Validated German versions of EQ-5D-5L and Kessler K-10 were available. The remaining text of the survey was translated from Australian English into German by native speakers of the target language who were fluent in English. The translation procedure included forward and backward translations as well as feedback from in-country persons.

### Sample

Online samples of approximately 1000 adults from the German general population were recruited both for Germany 1 and 2 surveys. The survey was sent out as a weblink for the respondents to complete at their leisure. Potential respondents are members of an online panel of persons willing to complete surveys for small payment. Participants were eligible if aged 18 to 80 years. Representativeness of the sample was investigated by comparison with national census data [22]. As the age and sex distribution in the Germany 1 sample differed considerably from that in the general population, quota sampling by age and sex was used in the Germany 2 valuation to achieve representativeness for these variables.

Additional sources had to be accessed to obtain estimates the population prevalence of chronic diseases in persons aged 18–80, as no differentiation within the group of persons aged 65+ was made in the official statistics. Estimates were derived indirectly from articles on the prevalence of chronic diseases in the elderly in Germany [23] and Sweden [24].

### Sample size considerations

Determination of sample size was based on the width of the confidence intervals (CI) for the utility decrements found in the Australian valuation study with a total of 1833 respondents. Using the notation [*u* − *d*, *u* + *d*] for 95% CIs of utility decrements (where *u* is the estimated utility decrement and *d* is half the width of the CI or the margin of error), all values of *d* in the Australian study were ≤ 0.031, and for all domains but one d was below 0.0245. For the German valuation study, we wanted to ascertain that utility weights can be estimated with *d* ≤ 0.05 throughout. When using a sample size of 1000 respondents and allowing for the possibility of a slightly larger spread due to a more heterogeneous response pattern (factor 1.2), the corresponding values are *d* ≤ 0.05 for all domains and *d* ≤ 0.0396 for all but one domain, i.e., the above condition regarding the error margin is satisfied.

### Statistical analysis

Statistical analyses were run using SPSS, version 24, and Stata, version 13. The latter software was used for advanced DCE analyses, in particular for the mixed logit model.

#### Testing representativeness and analyzing feedback questions

For comparison of the valuation sample with national statistics data with regard to socio-demographic and clinical characteristics, the Chi-square test was used. Feedback questions were analyzed by descriptive statistical methods.

#### DCE analyses

The main part of the analysis consisted of the determination of utility weights for the QLU-C10D using the approach proposed by [11]. The basic model for the utility of option *j* (scenario A or B) in choice set *s* for respondent *i* is given by

$$U_{isj} = \alpha {\text{TIME}}_{isj} + \beta X^{\prime}_{isj} {\text{TIME}}_{isj} + \varepsilon_{isj} ,$$

where TIME_*isj*_ is the survival time presented in option *j* and *X′*_*isj*_ is a set of dummies related to the levels of the corresponding health state. The errors ε_isj_ were assumed to be independent and identically Gumbel distributed. The parameters α (scalar) and β (vector) were estimated by conditional logistic regression. Regression weights were then converted into utility decrements consisting of the ratio of the health state parameters β and the time coefficient α to reflect the trade-off between health-related QOL and length of life [11]. This method was also used to analyze the Australian QLU-C10D valuation data [10]. To allow for correlated observations within respondents, a random subjects term was included in the model using generalized estimation equation (GEE) models with first-order autoregressive covariance structure. We also compared the utility weights of the complete sample with the utility weights obtained when omitting those respondents who perceived the DCE as difficult or very difficult (who may therefore have given less reliable replies). For this purpose, the mean absolute difference (MAD), the Pearson correlation coefficient, and the intra-class correlation coefficient (ICC) between the two sets of utility decrements were determined, both for Germany 1 and 2.

#### Comparison of utility decrements of the two German QLU-C10D versions

For this purpose, the DCE data of the two German samples were pooled. Comparison of the two versions regarding the effect of response level on utility decrements was performed by a series of likelihood ratio (LR) tests.

#### Modifications of DCE analysis and additional analyses

(i) Some of the utility decrements obtained in the analysis did not show a monotonic pattern, i.e., increasing severity coinciding with increasing decrements. When this occurred, the non-monotonic levels were combined. This restriction has been standardly imposed in previous studies [4, 10, 25].
(ii) As the distribution of age and gender in the Germany 1 valuation sample differed significantly from the German population, a weighted analysis was performed in addition to the unweighted one. For this, the sample was stratified by age group and sex, imposing weights to achieve representativeness for each combination of the two variables [19].
(iii) Additional analyses of the DCE data were performed by means of mixed logits, for both the Germany 1 and 2 datasets. In this model, it was assumed that coefficients α and β were drawn from a distribution, thus allowing for heterogeneous preference patterns between respondents. More details may be found in the paper on the Australian valuations [10]. As the mixed logit model deals with the distribution of parameters rather than with point estimates, its use for estimating utility decrements (which are derived taking ratios of α and β) entails considerable statistical problems. As noted by Gu et al., the distribution of the ratio can have an extremely wide spread when the denominator is close to zero (and the mean can be extremely high) [26]. Hence, we used the conditional logit model for estimating utility decrements, which is in line with the usual practice, but present the mixed logit results for those interested which dimensions demonstrate considerable preference heterogeneity.

## Results

### Complete cases and dropouts

An overview of the respondent flow is given in Appendix (Table 5). A proportion of those invited to the survey dropped out immediately, upon reading the description of the survey (14.3% and 10.0% for Germany 1 and 2, respectively); presumably it did not interest them. Further, for the Germany 2 survey, 34.4% were excluded as they were excess to quota sampling for their age and sex; there were no such exclusions for Germany 1, as there was no quota sampling. Of the remainder, completion rates were close to 90%. Specifically, 1002 of 1135 (88.3%) respondents entering the Germany 1 valuation component of the survey and 1016 of 1124 (90.4%) respondents meeting the quota-sampling criteria for the Germany 2 valuation completed all the survey components. These ‘complete case’ respondents form the analysis dataset for all results reported below.

### Socio-demographic and clinical data: comparison with national statistics

An overview of socio-demographic and clinical characteristics of the two valuation samples is given in Table 2. The Germany 1 sample showed significant departures from the general population in the distribution of respondents’ age, gender, and education. In particular, the sample included a smaller fraction of persons in the oldest age group, a smaller proportion of women, and a larger percentage of highly educated people compared to the German general population. As some of these differences were quite substantial (≥ 10%), we performed a weighted analysis of the DCEs in addition to the standard analysis. Quota sampling achieved population-representative distributions of age and sex for the Germany 2 sample, and it exhibited significant deviations from the general population only for education, again with a larger proportion of more highly educated persons.

**Table 2:** Distribution of socio-demographic and clinical characteristics—Germany 1 and Germany 2

Variable	Category	German census data^a^	Sample Germany 1 (*N *= 1002)			Sample Germany 2 (*N *= 1016)		
		%	*n*	%	Statistics	*n*	%	Statistics
Age	18–30	17.7	184	18.4	*χ*^2^ = 106.7, *p *< 0.001	175	17.2	*χ*^2^ = 1.10, *p *= 0.954
	31–40	15.3	168	16.8		165	16.2	
	41–50	18.6	220	22.0		182	17.9	
	51–60	19.8	244	24.4		206	20.3	
	61–70	14.3	155	15.5		144	14.2	
	71– 80	14.3	31	3.1^d^		144	14.2	
Gender	Male	49.5	610	60.9^c^	*χ*^2^ = 51.4, *p *< 0.001	498	49.0	*χ*^2^ = 0.09, *p *= 0.759
	Female	50.5	392	39.1^d^		518	51.0	
Education	Compulsory	37.4	251	25.0^d^	*χ*^2^ = 179.4, *p *< 0.001	295	29.0^d^	*χ*^2^ = 140.0, *p *< 0.001
	Lower secondary	31.4	246	24.6^d^		231	22.7^d^	
	Higher secondary	15.1	220	22.0^c^		219	21.6^c^	
	Tertiary (university, polytechnic)	16.1	285	28.4^c^		271	26.7^c^	
Marital status	Single	25.1	238	23.8	*χ*^2^ = 3.86, *p *= 0.277	266	26.2	*χ*^2^ = 7.38, *p *= 0.061.
	Married/partnership	61.1	613	61.1		584	57.5	
	Divorced/separated	10.5	122	12.2		125	12.3	
	Widowed	3.3	29	2.9		41	4.0	
Chronic diseases	Yes	36.7^b^	375	37.4	*χ*^2^ = 0.23 *p *= 0.634	402	39.6	*χ*^2^ = 3.59, *p *= 0.058
	No	63.3	627	62.6		614	60.4	

^a^Distribution of socio-demographic and clinical characteristics, restricted to an age range of 18–80

^b^Assuming a prevalence of 80% for chronic diseases in persons aged over 80 [based on prevalences given by Fuchs et al. [23] and Marengoni et al. [24]

^c^More than 5% points higher than in the German general population

^d^More than 5% points lower than in the German general population

### Feedback questions on the DCE

More than two-thirds of the respondents (Germany 1: 69.1%, Germany 2: 67.6%) regarded the presentation of the DCE as clear or very clear, and only a minority (12.1% vs. 11.5%) as unclear. However, almost half of the respondents (47.6% vs. 46.6%) considered the DCE task (choosing between situation A and B) as difficult or very difficult, only about a quarter (23.1% vs. 26.1%) found it easy or very easy. Even so, DCE results remained fairly stable when those respondents who perceived the task as difficult or very difficult were excluded from analysis. Thus, the MAD for the two sets of utility decrements (all respondents vs. those respondents who did not find the task difficult) was 0.0131 and 0.0178 for Germany 1 and 2, respectively. Pearson correlations between the two utility sets were *r* = 0.962 and *r* = 0.951, and ICCs were 0.957 and 0.940 for the two German versions. Regarding the response strategy used, almost half of the respondents replied that they concentrated on a few aspects or on those highlighted in yellow (45.6%, Germany 1 and 2 pooled), whereas 40.9% stated that they considered most or all aspects. Only a small fraction used other strategies (5.8%).

### Raw utility decrements (without correction for non-monotonicity)

**Fig. 1:**
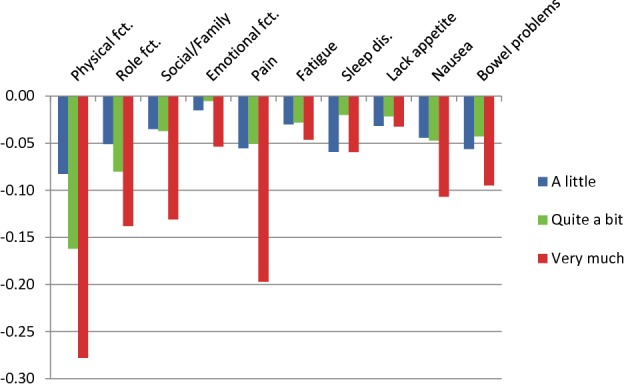
Utility decrements for the German version 1 of the QLU-C10D (raw decrements without adjustment for monotonicity)

**Fig. 2:**
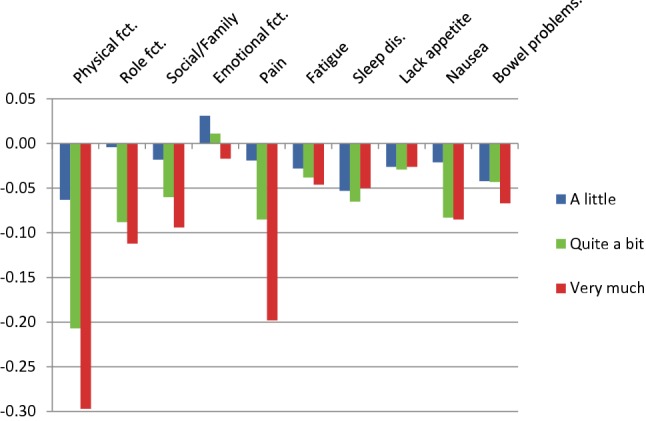
Utility decrements for the German version 2 of the QLU-C10D (raw decrements without adjustment for monotonicity)

Findings of the DCE analysis are shown in Figs. 1 and 2 and in Tables 3 and 4 (for Germany 1 and Germany 2, respectively). Numbers displayed are utility decrements for each dimension and severity level (a little, quite a bit, and very much). Note that utility decrements for the level “not at all” are 0 by definition. Utilities of the individual health states can be obtained by subtracting the respective utility decrements or a linear combination of them from 1.

**Table 3:** QLU-C10D utility decrements for German version 1 (Response level 3 = “mäßig”)

Parameter	Severity level	Parameter estimates^a^		Utility decrements		Utility decrements with imposed monotonicity	
		α	β	Estimate (β/α)	SE	Estimate	SE
Time		0.631					
Physical Functioning (restrictions)	2 (a little)		− 0.052	− 0.083**	0.020	− 0.084**	0.020
	3 (quite a bit)		− 0.102	− 0.162**	0.022	− 0.162**	0.021
	4 (very much)		− 0.176	− 0.278**	0.022	− 0.274**	0.021
Role Functioning (restrictions)	2 (a little)		− 0.032	− 0.051**	0.016	− 0.047**	0.016
	3 (quite a bit)		− 0.050	− 0.080**	0.016	− 0.078**	0.016
	4 (very much)		− 0.087	− 0.138**	0.015	− 0.134**	0.015
Social Functioning (restrictions)	2 (a little)		− 0.022	− 0.035*	0.015	− 0.035*	0.015
	3 (quite a bit)		− 0.024	− 0.037*	0.018	− 0.039*	0.017
	4 (very much)		− 0.083	− 0.131**	0.015	− 0.130**	0.015
Emotional Functioning (restrictions)	2 (a little)		− 0.009	− 0.015	0.015	− 0.013	0.014
	3 (quite a bit)		− 0.003	− 0.005	0.017	− 0.013	0.014
	4 (very much)		− 0.034	− 0.054**	0.014	− 0.054**	0.013
Pain	2 (a little)		− 0.035	− 0.055**	0.016	− 0.056**	0.014
	3 (quite a bit)		− 0.031	− 0.050**	0.018	− 0.056**	0.014
	4 (very much)		− 0.124	− 0.197**	0.015	− 0.196**	0.015
Fatigue	2 (a little)		− 0.019	− 0.030*	0.015	− 0.032*	0.013
	3 (quite a bit)		− 0.018	− 0.028	0.016	− 0.032*	0.013
	4 (very much)		− 0.029	− 0.046**	0.014	− 0.047**	0.013
Sleep	2 (a little)		− 0.037	− 0.059**	0.014	− 0.044**	0.013
	3 (quite a bit)		− 0.013	− 0.020	0.017	− 0.044**	0.013
	4 (very much)		− 0.037	− 0.059**	0.014	− 0.066**	0.013
Lacking appetite	2 (a little)		− 0.020	− 0.032*	0.014	− 0.029*	0.013
	3 (quite a bit)		− 0.014	− 0.022	0.016	− 0.029*	0.013
	4 (very much)		− 0.020	− 0.032*	0.014	− 0.034*	0.014
Nausea	2 (a little)		− 0.028	− 0.044**	0.015	− 0.043**	0.015
	3 (quite a bit)		− 0.030	− 0.047**	0.015	− 0.047**	0.015
	4 (very much)		− 0.067	− 0.107**	0.014	− 0.106**	0.013
Bowel problems	2 (a little)		− 0.036	− 0.056**	0.014	− 0.050**	0.013
	3 (quite a bit)		− 0.027	− 0.043**	0.016	− 0.050**	0.013
	4 (very much)		− 0.060	− 0.095**	0.013	− 0.095**	0.013

*p < 0.05, **p < 0.01

^a^Parameter estimates obtained from conditional logistic regression; α denotes the time parameter and β the health state parameters

**Table 4:** QLU-C10D utility decrements for German version 2 (Response level 3 = “ziemlich”)

Parameter	Severity level	Parameter estimates		Utility decrements		Utility decrements with imposed monotonicity	
		α	β	Estimate (β/α)	SE	Estimate	SE
Time		0.523					
Physical functioning (restrictions)	2 (a little)		− 0.033	− 0.063*	0.025	− 0.062*	0.025
	3 (quite a bit)		− 0.108	− 0.207**	0.025	− 0.201**	0.024
	4 (very much)		− 0.155	− 0.297**	0.024	− 0.290**	0.023
Role Functioning (restrictions)	2 (a little)		− 0.002	− 0.004	0.019	− 0.005	0.019
	3 (quite a bit)		− 0.046	− 0.088**	0.019	− 0.085**	0.018
	4 (very much)		− 0.059	− 0.112**	0.017	− 0.109**	0.017
Social Functioning (restrictions)	2 (a little)		− 0.009	− 0.018	0.017	− 0.019	0.017
	3 (quite a bit)		− 0.031	− 0.060**	0.019	− 0.059**	0.018
	4 (very much)		− 0.049	− 0.094**	0.016	− 0.093**	0.016
Emotional Functioning (restrictions)	2 (a little)		0.016	0.031	0.019	0.000	–
	3 (quite a bit)		0.006	0.011	0.020	− 0.007	0.016
	4 (very much)		− 0.009	− 0.017	0.017	− 0.029*	0.015
Pain	2 (a little)		− 0.010	− 0.019	0.018	− 0.019	0.018
	3 (quite a bit)		− 0.045	− 0.085**	0.019	− 0.082**	0.019
	4 (very much)		− 0.104	− 0.198**	0.017	− 0.195**	0.017
Fatigue	2 (a little)		− 0.015	− 0.028	0.017	− 0.027	0.016
	3 (quite a bit)		− 0.020	− 0.038*	0.018	− 0.037*	0.018
	4 (very much)		− 0.024	− 0.046**	0.016	− 0.047**	0.015
Sleep	2 (a little)		− 0.028	− 0.053**	0.017	− 0.050**	0.016
	3 (quite a bit)		− 0.034	− 0.065**	0.017	− 0.057**	0.014
	4 (very much)		− 0.026	− 0.050**	0.016	− 0.057**	0.014
Lacking appetite	2 (a little)		− 0.014	− 0.026	0.017	− 0.027	0.016
	3 (quite a bit)		− 0.015	− 0.029	0.018	− 0.029*	0.015
	4 (very much)		− 0.014	− 0.026	0.016	− 0.029*	0.015
Nausea	2 (a little)		− 0.011	− 0.021	0.017	− 0.023	0.017
	3 (quite a bit)		− 0.043	− 0.083**	0.017	− 0.082**	0.017
	4 (very much)		− 0.044	− 0.085**	0.016	− 0.085**	0.015
Bowel problems	2 (a little)		− 0.022	− 0.042**	0.016	− 0.044**	0.015
	3 (quite a bit)		− 0.022	− 0.043*	0.017	− 0.044**	0.017
	4 (very much)		− 0.035	− 0.067**	0.016	− 0.067**	0.015

*p < 0.05, **p < 0.01

^a^Parameter estimates obtained from conditional logistic regression; α denotes the time parameter and β the health state parameters

The largest utility decrements were observed for the domain of physical functioning (PF), with decrements of − 0.083, − 0.162, and − 0.278 for the three levels of restrictions in PF for Germany 1 and similarly sized decrements for Germany 2. For both German versions, the second largest utility decrements were seen for pain, followed by role functioning and social functioning. The decrements for the other domains were considerably lower, with nausea and bowel problems following in fifth and sixth place. Utility decrements were smallest for emotional functioning, fatigue, sleep disorders, and lacking appetite, attaining statistical significance only for a few severity levels.

### Constrained utility decrements

**Fig. 3:**
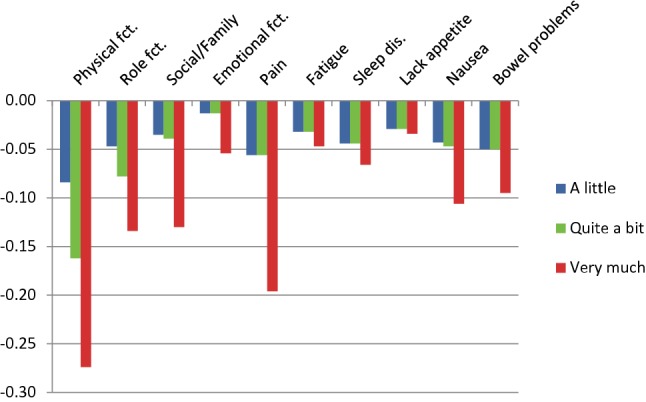
Utility decrements for the German version 1 of the QLU-C10D (with adjustment for monotonicity)

**Fig. 4:**
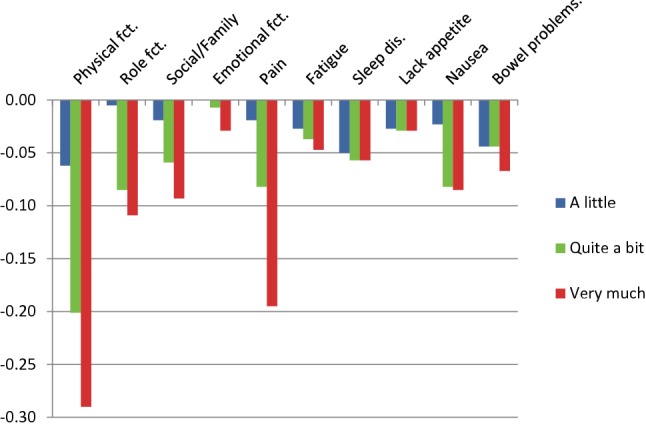
Utility decrements for the German version 2 of the QLU-C10D (with adjustment for monotonicity of levels)

**Fig. 5:**
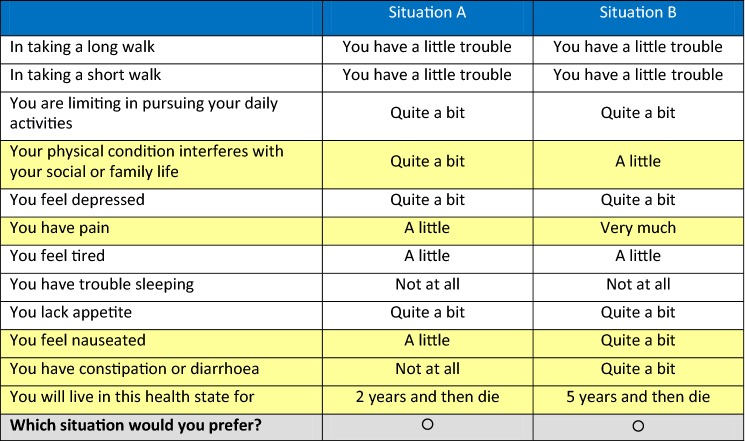
Example of a choice set used to determine utility weights for the QLU-C10D

In a total of six instances, the utility decrements for Germany 1 were not monotonically ordered (see Fig. 1). Most deviations from monotonicity were small and non-significant, only one reached statistical significance (for sleep disorders, *χ*^2^ = 5.76, *p* = 0.016). In all these cases, utility decrements for response level 2 (a little) and 3 (quite a bit) were reversed. To obtain a set of utility weights fulfilling the monotonicity condition, the analysis was rerun constraining levels 2 and 3 to a single utility decrement where required (see Fig. 3 and right-hand column of Table 3). For Germany 2, violations of monotonicity were observed in three cases, none of which reached statistical significance. In particular, no reversal of the levels 2 and 3 occurred. Monotonicity-constrained utility decrements for Germany 2 are shown in Fig. 4 and in the right-hand column of Table 4.

### Comparison of Germany 1 and 2

Likelihood ratio testing revealed a significant overall difference between the utility decrements of the two German versions (*χ*^2^ = 68.8, *df* = 30, *p* < 0.001). This was mainly due to an effect of response level 3, i.e., that level for which the wording of the two German versions differed (*χ*^2^ = 63.1, *df* = 10, *p* < 0.001). The corresponding coefficients all had negative signs indicating that the estimated utility decrements were larger for Germany 2 than for Germany 1 across domains, in accordance with our hypothesis. In addition, there was a significant effect of response level 4, “very much” (*χ*^2^ = 26.2, *df* = 10, *p* = 0.003). All but one of the beta coefficients had a positive sign showing that utility estimates for response level 4 were generally smaller for Germany 2 than for Germany 1. No significant interaction between level 2 and version was found. More details can be found in Appendix (Table 6).

### Calculation of QLU-C10D utilities

The basis for the calculation of QLU-C10D utilities both for Germany 1 and Germany 2 are the monotonically ordered utility weights as displayed in the right-hand side of Tables 3 and 4, respectively. Utilities are obtained by subtracting the respective utility weights from 1. For instance, the utility for the health state (23311 11111), i.e., little restriction in PF, quite a bit of restrictions in RF and SF, and optimal health in all other dimensions, amounts to

$$1 - 0.084 - 0.078 - 0.039 = 0.799,$$

in the metric of Germany 1 and to

$$1 - 0.062 - 0.085 - 0.059 = 0.794$$

in the metric of Germany 2.

The utility of the worst possible health state (4444444444) takes the value − 0.136 for Germany 1 and the value − 0.001 for Germany 2.

### Additional analyses

#### Weighted analysis for Germany 1

A *weighted analysis* of the DCE data for Germany 1, adjusting for non-representativeness with regard to age and sex, yielded very similar results as the unweighted analysis. With one exception the utility decrements of the weighted analysis differed from the original ones by less than 0.01 in either direction. A slightly larger difference was observed for level 3 of the SF domain (estimated decrements of − 0.037 and − 0.025 for the unweighted and weighted analyses, respectively, i.e., a difference of 0.012 points).

### Mixed logit analyses

Findings of the mixed logit analyses for Germany 1 and 2 are shown in Appendix (Tables 7, 8). Regarding non-monotonicity, basically the same patterns were found as for the conditional logistic regression analysis above. The majority of the estimated standard deviations of the model parameters were significantly greater than 0 both for Germany 1 (29 parameters of 31) and Germany 2 (27 of 31) reflecting considerable heterogeneity in individual respondents’ preferences.

## Discussion

The EORTC QLU-C10D is the first cancer-specific utility instrument for which valuations are being performed in multiple countries internationally using a standard valuation method. Germany is the second country after Australia [10] for which QLU-C10D utility weights become available. As the German version of the underlying parent QOL instrument is presently undergoing a revision of the wording of one response category, utility weights were determined for both versions (Germany 1 and 2).

Online samples were used for both valuations. Quota sampling by age and sex achieved representativeness for these two key demographic variables in the Germany 2 valuation survey. Representativeness of this sample was generally good for other characteristics, except for a surplus in respondents with high educational levels. Lack of quota sampling in the German 1 valuation resulted in significant non-representativeness for both age and sex. However, the effect of this imbalance on the derived utility decrements was small as shown by weighted analysis.

Generally, our findings on German QLU-C10D utility weights look plausible and agree with our expectations. In particular, DCE analyses resulted in a meaningful order of dimensions by size of utility weights. Physical functioning received the largest utility weights, followed by pain, role functioning, and social functioning. The cancer-specific dimensions of nausea and bowel functioning came in fifth and sixth place. This agrees well with the pattern found in the Australian QLU-C10D valuation where only one dimension, emotional functioning, received considerably larger utility weights than in our valuation [10].

Similar to the Australian QLU-C10D valuation, three dimensions particularly relevant to cancer patients were given fairly small utility weights: fatigue, appetite loss, and sleep disturbances. It may be that the relatively low utility decrements for these dimensions reflect a lack of experience of these symptoms in the general population. This calls for valuation studies to be performed in patient populations in order to scrutinize the above assumption. As a first step towards this aim, we have started with patient valuations for the QLU-C10D in Austria after completing valuations in the Austrian general population (not yet published).

Small utility weights were also observed for the dimension of emotional functioning. This may have to do with the German wording for the key item used to describe this dimension, “depressed.” It was translated as “niedergeschlagen” (similar to downcast or moody) in the QLQ-C30 which is probably perceived as weaker than the English word “depressed” by respondents.

Regarding monotonicity of utility weights, there were remarkable differences between the two German versions. While only a few small deviations from monotonicity were seen in the Germany 2 valuation, considerable problems with monotonicity occurred for Germany 1. All of these involved a reversal of the levels “a little” and “quite a bit” indicating that respondents had difficulties distinguishing these two response levels in the Germany 1 valuation task. Change of the German wording of the category “quite a bit” to a stronger expression obviously solved the problem as no reversal of these two categories occurred in the Germany 2 valuation.

A number of CUAs in oncology have been conducted in Germany or in multi-center studies including Germany in recent years. Some of these used generic utility instruments [27, 28], others obtained health utility values from the literature [29, 30], by expert ratings [31] or via mapping procedures [32]. The new utility instrument offers a valuable alternative as patient utilities can directly be obtained from the parent instrument, the QLQ-C30, which is routinely used in many oncological studies conducted in European countries, including Germany. Moreover, as a cancer-specific utility instrument it has the potential to capture cancer-specific treatment effects better than generic MAUIs like the EQ-5D, although this is yet to be tested empirically.

Our study has some limitations. First, there is the potential that our sample is non-representative of the entire population due to their self-selection into the online panel. Our quota sampling for age and sex ensured that our sample was representative on these two key demographics. However, there was an over-representation of respondents with high educational levels in both valuation surveys. This may be a typical characteristic of online samples as it was also found in the Australian survey [10]. It is important to note that the effect of education on health utility values has been found to be consistently small in a systematic review on EQ-5D valuation studies [33] and in other valuations [34, 35]. Second, some non-monotonicities were encountered in the Germany 1 survey. Consistent with the practice used by other researchers, we imposed constraints on model parameters to remove non-monotonicities [10].

In summary, the present paper provides utility weights for the new cancer-specific utility instrument, the QLU-C10D, for an economically important European country, Germany. This is of relevance for future cancer related CUAs performed in Germany. At the same time, the present paper is to be regarded as one piece of research within a larger program with a broader, international perspective. Thus, QLU-C10D valuations for a number of other countries, including the UK, France, Italy, Poland, Canada and the US, are presently underway. In their entirety they should provide a basis for more targeted decision making in cancer care.

## Appendix

See Fig. 5, Tables 5, 6, 7, and 8.

**Table 5:** Flow of study participants

Participants, exclusions and dropouts	Germany 1	Germany 2
Number invited/entered	1324	2021
Termination before start of survey		
Invited but declined to participate	26	26
Entered survey but quit after welcome page	163	176
Number excluded		
Over quota (age-by-sex)	0^a^	613
Response device too small (mobile phones, etc.)	0^b^	82
Participants continuing to valuation component of survey	1135	1124
Dropout before DCE (initial socio-demographics, QLQ-C30)	28 (2.5%)	41 (3.6%)
Dropout during DCE	96 (8.5%)	67 (6.0%)
Dropout after DCE (feedback questions, socio-demographics, EQ-5D, Kessler K-10)	9 (0.8%)	0 (0.0%)
Number of participants with complete DCE data	1002 (88.3%)	1016 (90.4%)

^a^Quota sampling (age, sex) was used only for Germany 2

^b^The device type used (desktop device, tablet, mobile phone, etc.) was only recorded for Germany 2

**Table 6:** Comparison of the two German versions of the QLU-C10D regarding effects of response level on utility weights

Model description	Effect tested	Model information		Comparison with full model (Likelihood ratio test)		
		*df*	*χ*^2^	Δ *df*	Δ *χ*^2^	*p* value
Full model (individual utility decrements for Germany 1 and Germany 2)	–	61	10037.7	–	–	–
Common utility decrements for Germany 1 and 2 throughout	Omnibus test of an effect of Version (Germany 1 vs. 2) on utility weights	31	9958.9	30	68.8	< 0.001
Common utility decrements for response level 3, individual decrements for levels 2 and 4	Interaction between response level 3 and Version (Germany 1 vs. 2)	51	9974.6	10	63.1	< 0.001
Common utility decrements for response level 2, individual decrements for levels 3 and 4	Interaction between response level 2 and Version (Germany 1 vs. 2)	51	10030.5	10	7.2	0.71
Common utility decrements for response level 4, individual decrements for levels 2 and 3	Interaction between response level 4 and Version (Germany 1 vs. 2)	51	10011.5	10	26.2	0.003

**Table 7:** Coefficient estimates from the mixed logit for Germany 1

Variable/domain	Level	Mean	Robust SE	SD	Robust SE
Duration	(Linear)	1.535	.062	.748	.029
Physical functioning	2	− .152	.022	.150	.025
	3	− .160	.025	.224	.026
	4	− .284	.023	.252	.023
Role functioning	2	− .105	.019	.084	.028
	3	− .098	.022	.196	.025
	4	− .181	.020	.156	.022
Social functioning	2	− .042	.020	.158	.022
	3	− .052	.020	.008	.029
	4	− .223	.020	.239	.021
Emotional functioning	2	.024	.020	.137	.030
	3	.033	.021	.158	.031
	4	− .100	.019	.170	.028
Pain	2	− .076	.020	.169	.026
	3	− .055	.021	.091	.037
	4	− .309	.022	.375	.026
Fatigue	2	− .006	.019	.173	.022
	3	− .037	.021	.134	.025
	4	− .097	.019	.089	.029
Sleep disturbances	2	− .015	.018	.060	.029
	3	.051	.022	.224	.024
	4	− .080	.019	.067	.034
Appetite loss	2	− .008	.019	.133	.024
	3	− .004	.021	.124	.032
	4	− .026	.019	.099	.028
Nausea	2	− .075	.019	.125	.028
	3	− .071	.020	.114	.025
	4	− .207	.020	.209	.025
Bowel problems	2	− .056	.019	.123	.026
	3	− .049	.021	.184	.031
	4	− .137	.019	.188	.024

**Table 8:** Coefficient estimates from the mixed logit model for Germany 2

Domain	Level	Mean	Robust SE	SD	Robust SE
Duration	(Linear)	1.314	.056	.735	.030
Physical functioning	2	− .100	.020	.134	.022
	3	− .233	.022	.136	.022
	4	− .252	.020	.178	.026
Role functioning	2	− .008	.019	.178	.027
	3	− .114	.021	.040	.050
	4	− .102	.019	.131	.021
Social functioning	2	− .033	.018	.088	.026
	3	− .086	.020	.146	.027
	4	− .127	.019	.172	.025
Emotional functioning	2	.067	.018	.143	.031
	3	.028	.019	.169	.026
	4	− .047	.018	.108	.039
Pain	2	− .028	.017	.083	.032
	3	− .113	.019	.047	.037
	4	− .234	.019	.250	.026
Fatigue	2	− .028	.018	.170	.026
	3	− .047	.019	.154	.025
	4	− .084	.017	.009	.062
Sleep disturbances	2	− .055	.018	.118	.025
	3	− .071	.020	.163	.035
	4	− .068	.017	.146	.033
Appetite loss	2	− .005	.017	.118	.028
	3	− .029	.019	.119	.029
	4	− .031	.018	.161	.033
Nausea	2	− .006	.018	.075	.054
	3	− .090	.019	.073	.032
	4	− .108	.018	.121	.026
Bowel problems	2	− .025	.016	.101	.027
	3	− .025	.018	.126	.023
	4	− .070	.017	.120	.029
